# A Molecular Dynamics Study on Rotational Nanofluid and Its Application to Desalination

**DOI:** 10.3390/membranes10060117

**Published:** 2020-06-06

**Authors:** Qingsong Tu, Wice Ibrahimi, Steven Ren, James Wu, Shaofan Li

**Affiliations:** 1 Civil and Environmental Engineering, University of California, Berkeley, CA 94720, USA; howietu@berkeley.edu (Q.T.); mwibrahimi@berkeley.edu (W.I.); 2 Amador Valley High School, Pleasanton, CA 94566, USA; hr1646@pleasantonusd.net; 3 Granada High School, Livermore, CA 4550, USA; jjwu1@yahoo.com

**Keywords:** reverse osmosis desalination, graphene membrane, molecular dynamics, nano-porous materials, rotational centrifuge

## Abstract

In this work, we systematically study a rotational nanofluidic device for reverse osmosis (RO) desalination by using large scale molecular dynamics modeling and simulation. Moreover, we have compared Molecular Dynamics simulation with fluid mechanics modeling. We have found that the pressure generated by the centrifugal motion of nanofluids can counterbalance the osmosis pressure developed from the concentration gradient, and hence provide a driving force to filtrate fresh water from salt water. Molecular Dynamics modeling of two different types of designs are performed and compared. Results indicate that this novel nanofluidic device is not only able to alleviate the fouling problem significantly, but it is also capable of maintaining high membrane permeability and energy efficiency. The angular velocity of the nanofluids within the device is investigated, and the critical angular velocity needed for the fluids to overcome the osmotic pressure is derived. Meanwhile, a maximal angular velocity value is also identified to avoid Taylor-Couette instability. The MD simulation results agree well with continuum modeling results obtained from fluid hydrodynamics theory, which provides a theoretical foundation for scaling up the proposed rotational osmosis device. Successful fabrication of such rotational RO membrane centrifuge may potentially revolutionize the membrane desalination technology by providing a fundamental solution to the water resource problem.

## 1. Introduction

Clean water resources are essential to the survival of human life and human civilization. Developments of new techniques for water treatment and desalination have been a focal point for both sustainability research and environmental engineering [1]. In the past several decades, the membrane-based reverse osmosis (RO) technologies have been more prevalent and have accounted for more than 40 percent of the total desalination capacity [2], due to its energy efficiency compared to other desalination methods [3], such as thermal-based multi-stage flash method [4] and electrodialysis method [5]. Among all membrane materials, nanomaterials have many advantageous physicochemical properties that make them particularly attractive as separation media for water purification [6,7,8,9,10,11].

Recently, exciting progress has been made in the developments of carbon-based membrane materials and structures (such as Graphene, Graphene Oxide (GO) and Carbon Nanotube (CNT) [12,13,14,15,16]), which are identified to be excellent candidates for RO desalination because of their porous microstructures and excellent ion rejection abilities. Graphene membranes have excellent impermeability to molecules as small as helium [12,13] and a high water flux rate because of the property of hydrophobicity [14]. Surwade et al. manufactured a nanoporous graphene (NPG) membrane and observed that water molecules could pass through the membrane quickly with a very high salt rejection rate by controlling the radius of the nanopores on the NPG membrane [17]. Grossman theoretically investigated graphene membrane desalination and found NPG can not only enhance the rejection of ions, but also improve the permeation of water [18,19]. Graphene Oxide (GO) membranes are believed to be suitable alternative materials to overcome problems existing in pristine Graphene membranes. The existence of functional groups (such as hydroxyl, epoxy and carbonyl groups) [20,21] makes the GO membrane more hydrophilic and more accessible for synthesis. Hummer et al. studied water molecules flowing through CNT membranes and observed a high flow rate [15], which was verified by MD calculation showing that water flux rate inside the CNTs can be several magnitudes higher than that in the open environment [16].

However, several inherent drawbacks from the materials mentioned above hinder their practical applications in real desalination systems. First, self-contamination (fouling) of the membrane material can significantly degrade the efficiency of desalination. The fouling problem commonly exists in almost all current RO membrane methods, due to the high salt concentrations around nanopores and complicated physicochemical interactions between membrane materials and solutions [22,23]. One of the main reasons for fouling is initiated from the applied high hydrostatic pressure in the conventional desalination procedures [24]. Although this high hydraulic pressure on the porous membrane is necessary to balance osmotic pressure and maintain high freshwater flux [18], it also facilitates the blockage of pores on the membrane by pushing more ions inside it. Second, the high hydrostatic pressure on top of the membrane can decrease the interlayer distance between a layered membrane material. The narrower channels inside the membrane make it harder for water molecules to flow through the membrane and therefore decrease the filtration efficiency. Meanwhile, the high pressure can potentially fracture the membrane, which means the selected membrane material has to satisfy mechanical stability. Finally, although CNT membranes are natural porous materials, it is problematic to select suitable diameters of CNTs so that only water molecules can pass through while other ions are blocked. It is also challenging to align and assemble such CNTs into membranes and use them in real experiments.

**Figure 1:**
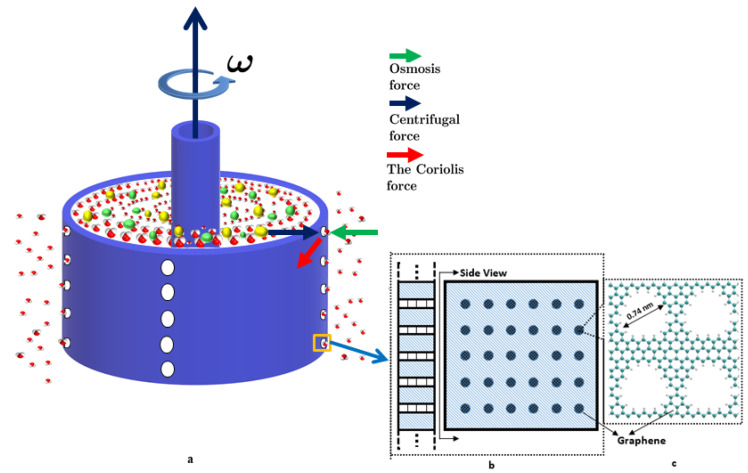
Proposed scaled up rotational nanofluidic device with nanoporous membrane wall. (**a**) Schematic illustration of an inner rotator generated swirling motion, (**b**) The coarse scale holes on the wall of the centrifuge, and (**c**) the fine scale pores in the graphene membrane patch covered on the coarse sale holes in the centrifuge wall.

In order to minimize the fouling problem, we propose a nanofluidic device—a rotating system with a nanoporous membrane, motivated by a centrifuge. A scaling-up strategy to fabricate such a nanofluidic device at the macroscale has previously been proposed in [25]. By utilizing the advantages of Couette flow around the nanoporous wall of the device, we theoretically proved that the issues mentioned above can be resolved and a higher desalination efficiency can be achieved. As shown in Figure 1 and in Appendix B, holes with mesoscale sizes are created on the wall of the macroscale size container, which separates the fluid into two reservoirs. The first holding saltwater inside the container and the second holding the pure water outside the container. The mesoscale holes are on the order of micron, which are filled with holes on the micro-scale that are in turn filled with nanopores.

Nanoporous membrane materials (such as Graphene or GO) are filled in the mesoscale holes with sub-nanometer pores on the porous material. An osmotic pressure $P_{os}$ pointing inward of the container is developed at the interface of fresh water and salt water, because of the concentration difference of ions between them. Naturally, water molecules will flow into the container because of this osmosis pressure; however, when a rotator inside the centrifuge rotates with an angular velocity ω, a centrifugal force will be developed among the ions and the water molecules. The centrifugal force grows larger with larger ω. Reverse osmosis will happen if the angular velocity ω is large enough so that the developed “centrifugal pressure” $P_{ext}$ (centrifugal force per unit area) is greater than the osmotic pressure: $\Delta P=P_{ext}-P_{os}>0$. The net outward pressure ΔP will drive the water solution inside the container to flow outward, but the finite sized pores will block the ions from exiting.

In this work, we use the classic molecular dynamics (MD) method to model and simulate such a rotating nanoporous structure at the submicron scale. Its scaled-up macroscale model is also analyzed by applying conventional fluid dynamics theory in a comparison study. Both the nanoscale and the macroscale structure are studied and compared. This paper is structured as followed: In the Results section, velocity profiles, slipping properties, and Taylor instability are investigated with data from the MD simulations. The relationship between angular velocity, equivalent pressure, and volumetric flow is discussed. The spiral motion of salt ions is provided as evidence for preventing fouling with the current model. Theoretical analysis based on Couette flow theory is employed to link the ion transportation through the nanoporous membrane with pressure profiles and volumetric flow rates. In the Discussion section, key findings from the results are summarized and the possibility of scaling up the model to a macroscale system is discussed, which can be used in real engineering desalination or water purification practice.

## 2. Results

### 2.1. Model Description

**Figure 2:**
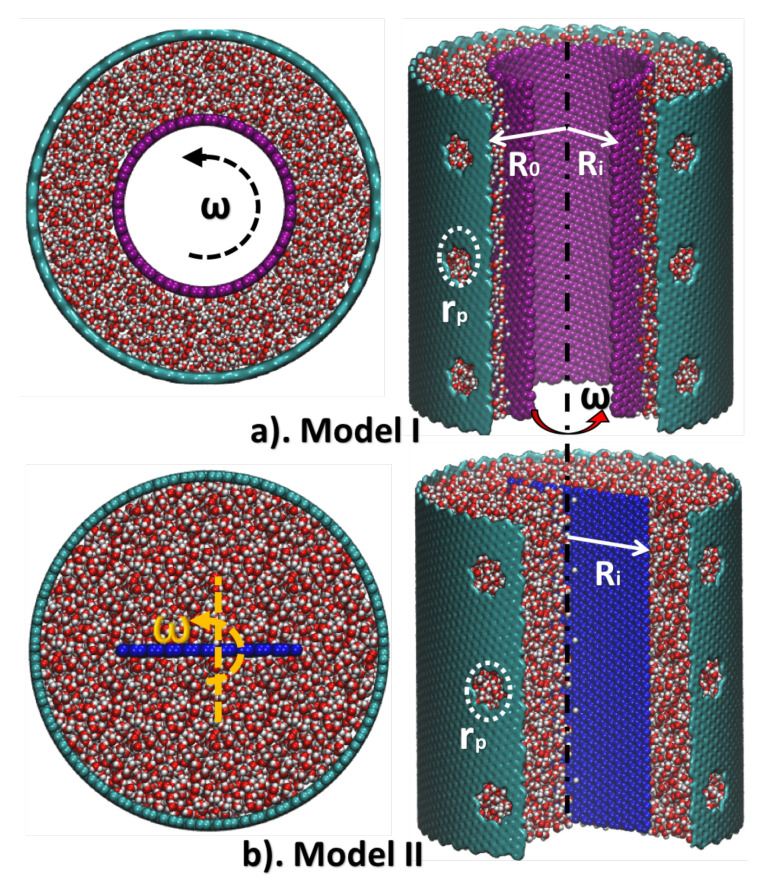
Molecular structures of two types of models for MD calculations. (**a**) The top and bisection view of Model I, with light-blue tube represents nanoporous membrane and pink tube represents rotator. (**b**) The top and bisection view of Model II, with light-blue tube represents nanoporous membrane and dark-blue tube represents rotator.

The molecular models are shown in Figure 2. Two different models are built to study the effect of two different boundary conditions on the rotational fluid fields. In both models, the outer-layer large tubes are a nanoporous membrane (such as Graphene or GO) with different sizes and numbers of pores. The rotator that generates a rotational fluid field and develops the centrifugal force is modeled as a small tube in Model I, and a blade in Model II. Notably, for simplicity, hydrophobic materials (Carbon Nanotube for Model I and Graphene for Model II) are used here in construction and modeling of rotator. In the real application, hydrophilic materials are preferable for the rotator in order to increase the friction between the fluid and the rotator, while hydrophobic materials are suitable for the porous membrane. However, for comparison purposes, we build a rotating blade in Model II in order to generate as large a rotating fluid as possible, which functions similar to the behavior of a rotator made of hydrophilic material.

In all MD calculations, outer-layer tubes have the same radius $R_{0}=4.2$ nm and same length $L_{0}=9$ nm. On the wall of the outer-layer tubes, twelve pores with radius $r_{p}=0.42$ nm are created. (Only six pores are shown in Figure 2, and other pores may be seen when rotating the centrifuge). The size of pores is selected according to the suggestion in the literature e.g., [26,27], so that water molecules can flow through the membrane freely while ions are blocked. The inner-layer tube in Model I and the blade in Model II have the same radius $R_{i}=2$ nm with varying angular velocity ω, to generate different fluid motions. However, the mechanisms of the generated rotating field are different. In Model I, the water swirling motion is generated by the viscous force of the rotation of inner-layer tube, whereas in Model II the rotation is generated by a direct propelling force exerted by the inner blade. Fluid velocity in Model I will be smaller than that in Model II under the same angular velocity applied on the two types of rotators. Models are solvated into water boxes with size 10 nm × 10 nm × 9 nm. The salinity of seawater is around 35 grams/liter (corresponds to a NaCl concentration of ΔC=0.6 M), which can be equivalently converted into 66 pairs of Na${}^{+}$ and Cl${}^{-}$ ions with a total number of 9770 water molecules in Model I, and 96 pairs of Na${}^{+}$ and Cl${}^{-}$ ions with a total number of 14,110 water molecules in Model II.

### 2.2. Motion of Water Molecules and Ions

**Figure 3:**
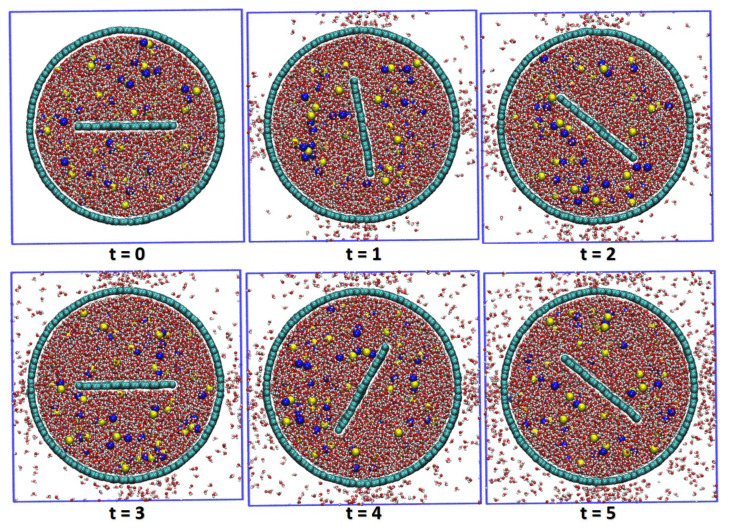
Snapshots of Model II over six simulation times, in unit nano-second. Na${}^{+}$ and Cl${}^{-}$ ions are in blue and yellow color, other molecules are H${}_{2}$O with oxygen atom in red color.

Snapshots from a typical run of Model II are depicted in Figure 3. Water molecules that are initially outside the nanoporous membrane are not shown here to highlight the flow of water molecules from inside to outside of the membrane. A constant angular velocity ω is applied on the blade so that all molecules and ions rotate with the rotator. As time progresses, water molecules gradually flow out from inside the membrane to the outside through pores on the wall while Na${}^{+}$ and Cl${}^{-}$ ions are blocked inside because of the sub-nanometer pores. An appreciable distance of water molecules and ions to the membrane (∼0.2 nm) is observed, which is because of the hydrophobic property of the membrane material. Notably, the hydrophobicity of the membrane can be beneficial in two aspects. First, less friction of the membrane to water molecules enables a larger centrifugal pressure $P_{\omega }$. Second, there is little friction force between the hydrophobic graphene membrane and hydrated ions, so that it enables a fast boundary slip motion of the ions, which reduces the fouling of the membrane by the hydrated ions.

**Figure 4:**
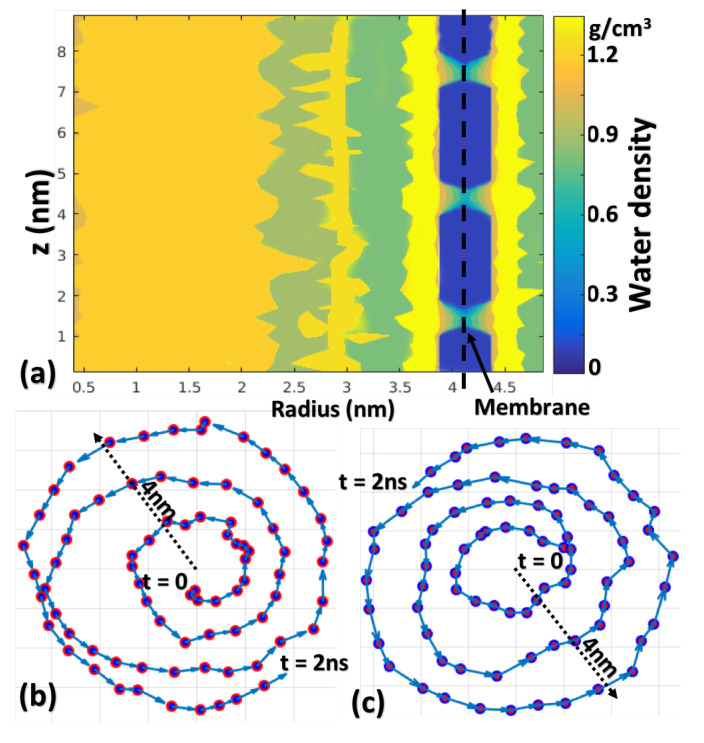
(**a**) Water density profiles of model type II along the radial and longitudinal directions under ω=17.5 rad/ns (Values along azimuthal direction are averaged). The trajectory of a typical Na${}^{+}$ ion (**b**) and Cl${}^{-}$ ion (**c**) which are located near the center at t=0 and move to the membrane wall at t=2 ns.

The water density profile computed is shown in Figure 4a, in which the blue area (3.9 nm < *r* < 4.4 nm) indicates zero water density, corresponding to the impermeable wall occupied by carbon atoms. The three channels correspond to the three nanopores on the membrane. The layered structure of water molecules along the radial direction is observed here, which was also reported in other works e.g., [28]. Among different mechanisms, two of the possible causes for the layered structure of water molecule distribution are the molecular attraction of the membrane and the centrifugal force during rotational motion. The first peak is located near the membrane at 3.6 nm < *r* < 3.9 nm and 4.4 m < *r* < 4.7 nm, with magnitude ∼1.5 times larger than bulk water. The second peak is located at 2.8 nm < *r* <3.1 nm with magnitude ∼1.2 times larger than bulk. The three valleys between three peaks are located at 2.4 nm < *r* < 2.8 nm, 3.1 nm < *r* < 3.6 nm and 4.7 nm < *r* < 5 nm with magnitude ∼0.8. The peaks and valleys in the water density profile are noted to better describe the behavior of the nanofluid in this rotational device, because they show where the water molecules are most likely to be found inside the centrifuge. Notably, the density of water molecules increases in the radial direction due to the centrifugal force.

Figure 4b,c display the trajectory of Na${}^{+}$ and Cl${}^{-}$ ions from the center to the nanoporous membrane. The spiral shape trajectories indicate that ions are affected by the rotational field. A centrifugal force is developed on these ions and water molecules because of the rotational fluid field which facilitates their radial motion. Some water molecules near pores might flow out of the container under this centrifugal force while all ions will continue rotating near the inner wall of the nanoporous membrane. As mentioned in the previous section, one of the main advantages of this system is the self-assisted cleaning mechanism by preventing the blockage of nanopores from ions. Although the centrifugal force exerted on ions near the entrance of pores can push them into the filtration channels and cause blockage, which is the main reason for fouling in classic RO methods, the unique tangential force in the rotating fluid field, which is mainly attributed to the Coriolis force, may flush these ions away from the surface of the membrane and hence the nano channels. This dynamic motion of ions will ensure the clearance of most nanopores and the continuous transport of water molecules from inside to outside through those active pores.

### 2.3. Azimuthal Velocity and Boundary Slipping

**Figure 5:**
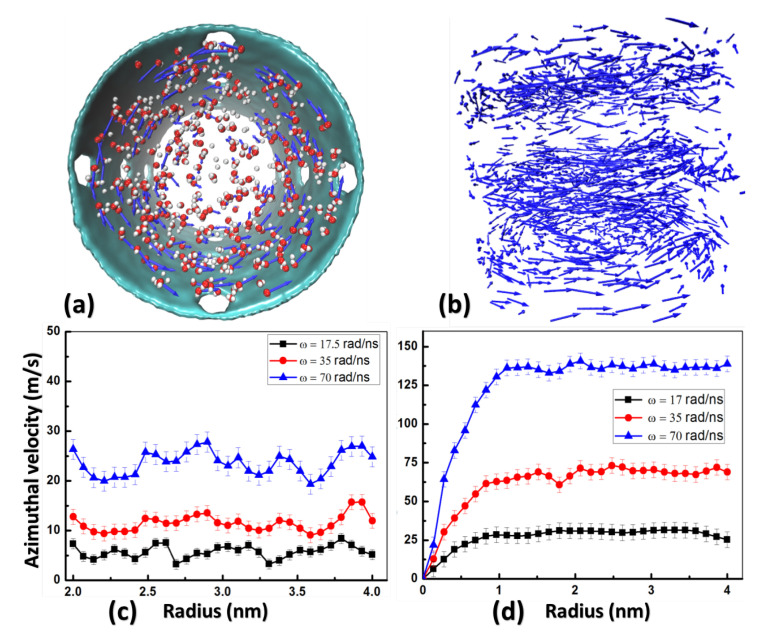
(**a**) Top-view of rotating water molecules (Oxygen in red and Hydrogen in white) with velocity vector (blue arrows) plotted on Oxygen. (**b**) 3D plot of velocity field of water molecules. Only velocity vectors (blue arrows) are shown. Azimuthal velocity $v_{\theta }$ along radial direction of Model I (**c**) and Model II (**d**) under different angular velocities.

Snapshots of velocity vectors of water molecules for Model II are depicted in Figure 5a,b. The average distributions of the azimuthal velocity $v_{\theta }$ along the radial direction for all water molecules of Model I and Model II are shown in Figure 5c,d respectively. During MD calculations, high angular velocities ω ranging from 0.1 rad/ns to 100 rad/ns are applied to rotators, which can hardly be achieved in real macro-scale experiments. However, with high angular velocity ω, MD calculations can speed up and more data can be collected for analysis to reduce statistic errors. A similar approach has been employed and verified by many other similar MD calculations to save computational resource [29,30]. In addition, this approach can be justified by our data from MD calculations and derivations from fluid dynamics that water flux scales linearly with net pressure. This means relations obtained from this work can be used to extrapolate results under lower angular velocities used in the real system.

Although rotators of Model I and Model II have the same tangential velocity at the inner radius $r=R_{i}$ under the same angular velocity ω with value $\omega R_{i}$, the rotating fields of water generated by them are entirely different, as shown in Figure 5c,d. Within the range of 0 < *r* < 2 nm, the azimuthal velocity $v_{\theta }$ of Model I is zero (0<r<2 nm excluded from plot in Figure 5c) because nothing exists inside the cylinder rotator, while $v_{\theta }$ of Model II increases almost linearly (Figure 5d) because any water molecules and ions within this range (0<r<2 nm) can be accelerated by ω of the blade rotator. Within the range of 2 nm < *r* < 4 nm, water molecules in both models have almost constant $v_{\theta }$, but different magnitudes under different angular velocities. In general, the azimuthal velocity $v_{\theta }$ of Model II is much larger than that of Model I, and both are smaller than their tangential velocity at the inner radius, $\omega R_{i}$. Note that the azimuthal velocity near the CNT walls increases with larger angular velocity ω. Multiple rotor velocities were analyzed to observe this behavior. It is also notable that only the most extreme ω in Figure 5d leads to an azimuthal velocity close to 150 m/s, while in a Maxwell distribution the mean molecular velocity of H${}_{2}$O at 20 degrees centigrade is about 595 m/s [31].

**Figure 6:**
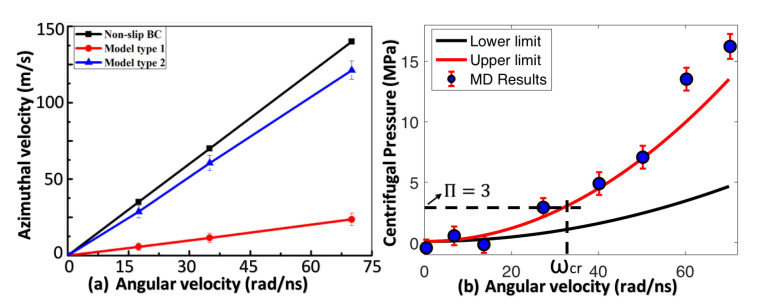
(**a**) Averaged azimuthal velocities of fluid near $r=R_{i}$ as a function of angular velocity ω for Model I and Model II. The curve “Non-slip BC” means $v_{\theta }=R_{i}\omega$. (**b**) Lower and Upper limits of the centrifugal pressure $P_{\omega }$ derived from fluid dynamics and that obtained from MD calculations.

A slipping parameter $\alpha_{i}$ can be defined here to describe the rotating of fluid caused by the rotator: $\alpha_{i}=v_{\theta }\left(R_{i}\right)/\omega R_{i}$, where $v_{\theta }\left(R_{i}\right)$ is the azimuthal velocity of fluids at the inner radius $r=R_{i}$. From Figure 5c,d, we can get the slipping parameter $\alpha_{i}$ for both Model I and Model II. Averaging data in range 3 nm < *r* < 4 nm for each curve in Figure 5c,d and plotting as a function of the angular velocity of rotators (as shown in Figure 6a), we find that $\alpha_{i}=0.16$ for Model I and $\alpha_{i}=0.82$ for Model II. The small value of $\alpha_{i}$ in Model I means friction between water molecules and the surface of the carbon nanotube (served as a rotator in Model I) are very small, which agrees well with other studies on the slip properties of Graphene/CNT/GO membranes [7,32]. However, while most of these studies focused on the unimpeded water transport in basal CNT nanochannels, we discovered that a slight friction still exists between the porous graphene membrane and water molecules during rotational motions. As discussed later, a no-slip boundary condition is preferred ($\alpha_{i}\to 1$) for fluid near the rotator, therefore, Model II is a better choice than Model I. Notably, changing the material of rotator in Model I to more hydrophilic material can also increase $\alpha_{i}$, such as GO and MoS${}_{2}$ which show much lower slipping property than carbon nanotube.

### 2.4. Derivation of Centrifugal Pressure

Following the classic fluid dynamics theory, we can derive the centrifugal pressure $P_{\omega }$ required to balance the osmotic pressure, as shown in the Method section. Since boundary conditions in Model I and Model II corresponds to the maximal and minimal slipping situation respectively, we can obtain the lower and upper limits of the centrifugal pressure $P_{\omega }$ by substituting the slipping parameter $\alpha_{i}$ into Equations (5) and (6),

$$P_{\omega }^{Low}=0.1+9.30\times 10^{-4}\omega^{2}\;\left[MP_{a}\right],\;\;\mathrm{and}\;\;P_{\omega }^{Up}=0.1+2.74\times 10^{-3}\omega^{2}\;\left[MP_{a}\right]\;.$$

The upper and lower limits of the centrifugal pressure correspond to the two idealized boundary conditions from fluid dynamics theory, which is further discussed later. The results from MD calculations can be compared to the upper and lower theoretical limits of centrifugal pressure to better understand how fluid dynamics theory relates to the nanoscale. Note the water density ρ used here is 1.5 g/cm${}^{3}$, based on results in Figure 4a. Meanwhile, the centrifugal pressure $P_{\omega }$ can be obtained from MD calculations by summing up the radial force ($F_{ir}$) of all water molecules within 0.3 nm (thickness of the first water layer) of the nanoporous membrane and then divided by the area of the membrane ($A_{m}$). Centrifugal pressure obtained from both fluid dynamics and MD simulation are plotted in Figure 6b with $\alpha_{i}=0.82$ from Model II.

The black and red lines in Figure 6b correspond to the lower and upper limits of the centrifugal pressure $P_{\omega }$ derived from classic fluid dynamics. MD results are plotted in blue dots. From Figure 5d, the value of $v_{\theta }$ is almost a constant from the inner radius $r=R_{i}$ to the outer radius $r=R_{o}$ under the same angular velocity ω, which means the boundary condition for fluid near the nanoporous membrane is almost ideal-slip (with outer slipping parameter $\alpha_{o}\approx \alpha_{i}$). Therefore, it is expected that the centrifugal pressure $P_{\omega }$ acquired from MD calculations (notated as $P_{\omega }^{MD}$) would be close to the upper limit $P_{\omega }^{Up}$ where $\alpha_{o}=\alpha_{i}$. Meanwhile, $P_{\omega }^{MD}\leq P_{\omega }^{Up}$ should always hold if the classic fluid dynamics theory is still valid at the nanoscale. However, comparing $P_{\omega }^{MD}$ with $P_{\omega }^{Up}$ in Figure 6b, we discover MD results agree well with the upper limit at relatively smaller angular velocity ω but start to deviate when ω becomes larger. The deviation is probably from the Taylor instability (such as turbulence) which will be discussed later. With these results, we conclude that the modified fluid dynamics theory (by incorporating slipping property of boundaries) is still valid at the nanoscale before the fluid becomes unstable under large ω.

From Figure 6b, we can also obtain the critical angular velocity $\omega_{cr}$ with value 32.55 rad/ns (the vertical dashed line) needed to balance the osmotic pressure with value Π = 3 MPa (the horizontal dashed line), which is the threshold value for water filtration. Water molecules can flow out from pores on the nanoporous membrane only when the angular velocity ω of the rotator in Model II is higher than $\omega_{cr}$.

### 2.5. Taylor Instability Due to High Rotational Flux

**Figure 7:**
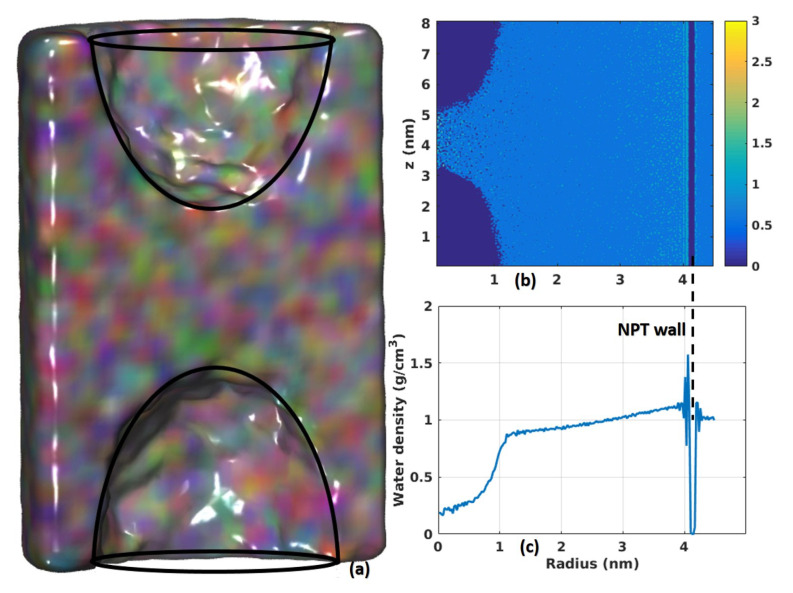
Taylor instability and vortex when ω=90 rad/ns. (**a**) Two vortexes (sketched by black lines) are observed inside the container. The fluid is rendered as colored colloid for better 3D view. (**b**) Water density profile in radial-longitudinal plane. (**c**) Distribution of water density along radial direction. The Nano-Porous Tube (NPT) wall in the figure signifies the location of the CNT membrane wall and that its properties are held at constant over time.

Higher water flux can be reached by increasing the angular velocity ω of the rotator. However, we may encounter other problems as ω becomes too large. First, a larger centrifugal force might be developed. This force can push ions more in-depth into the nano-channels causing blockage or even damage to the membrane material. It is also possible that ions are “squeezed out” from the inside leading to degradation of the rejection rate. Second, the Taylor-Couette instability can be developed when Reynolds’ number becomes significant. We note that once the angular velocity of the rotator exceeds a specific value, hydrodynamic instability and turbulence will occur, which may affect the efficiency of the desalination process, as shown in Figure 7.

Two typical vortexes symmetric to the central plane can be seen inside the model when ω≥70 rad/ns. Figure 7a shows the shape of the vortex and Figure 7b shows the density of water molecules along radial and longitudinal directions, with the azimuthal direction averaged out. The dark blue regions correspond to the two vortexes in Figure 7a. A more detailed analysis of water density is shown in Figure 7c, a quadratic shape is observed in the vortex area when both azimuthal and longitudinal directions are averaged out. Outside the vortex area, water density increases linearly to around 1.2 g/cm${}^{3}$, and then follows the layered structure when approaching the membrane, as discussed in Figure 4a. However, the width of the layered water structure here is much smaller than that in Figure 4a, because of the squeezing effect from large angular velocity ω. Further study is needed to investigate the phenomenon of Taylor instability at the nanoscale.

## 3. Discussion

**Figure 8:**
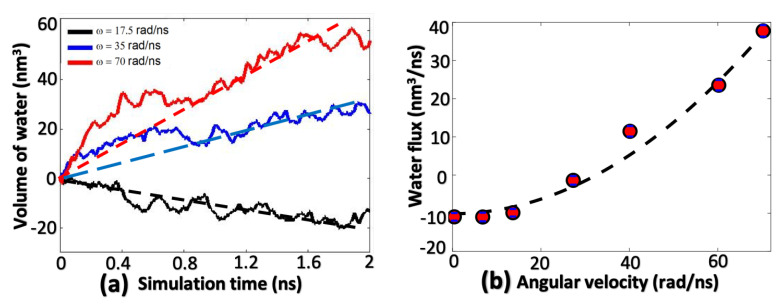
(**a**) Volume of filtrated water as a function of simulation time. (**b**) Water flux as a function of angular velocity.

One of the most significant features of the present system regards the angular velocity dependence of water flux. Figure 8a shows the total volume of water molecules flowing out from inside of the container as a function of simulation time (assuming one water molecule occupies a volume ≈$3\times 10^{-2}$ nm${}^{3}$). Linear interpolating of the three curves under three angular velocity ω is shown in dashed lines for the calculation of water flux. When $\omega <\omega_{cr}$, the centrifugal pressure $P_{\omega }$ is smaller than the osmotic pressure Π, leading to an inward net flow of water molecules (corresponds to the negative slope of black curves). When $\omega >\omega_{cr}$, the net water flow becomes outward (positive slopes of red and blue curves) and has a larger slope when ω is larger. Water flux (water flow per time) are the slopes of dashed lines in Figure 8a, which are plotted as a function of angular velocities ω in Figure 8b. Water flux predicted from MD calculations is a quadratic function of angular velocity ω, which agrees well with Equation (9) derived from fluid dynamics. Recall that pressure is also quadratically proportional to ω (Figure 6b), therefore we can conclude that water flux is linearly proportional to the overall centrifugal pressure as reported in other studies [32]. The centrifugal pressure in their works is from hydrostatic pressing while in our work it is from the centrifugal motion of the rotational fluid.

**Figure 9:**
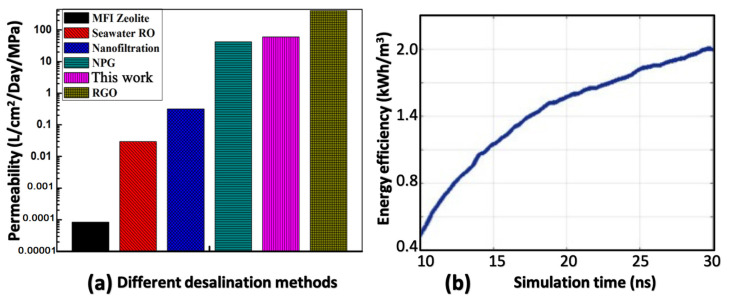
(**a**) Comparison of permeability of the current system with other RO materials. (**b**) Calculated energy efficiency as a function of simulation time, under ω=35 rad/ns.

Since we already proved fluid dynamics theory is still valid at the nanoscale, MD data in Figure 8b are fitted with Equation (9) to obtain the permeability of this system, which is 60 (L/cm${}^{2}$/day/MPa). A comparison between the permeability of the proposed device and the experimentally measured values from Pendergast et al. [33] and Grossman et al. [18,19,26] is shown below in Figure 9a. The water permeability of the proposed system is much better than three of them and comparable to the best two materials, which means that the desalination efficiency of this system could be 3–4 orders of magnitude higher than that of commercially available RO devices.

Energy efficiency of this system is also estimated with the relation: $\eta =\frac{Energy\;Input}{Filtrated\;Water}$. EnergyInput is defined as the kinetic energy change of the system within a time period Δt, and FiltratedWater is defined as the total number of water molecules passing through the nanoporous walls within the same time Δt. A longer duration MD simulation (>30 ns) is conducted with applied angular velocity ω=35 rad/ns. Figure 9b shows the value of energy efficiency as a function of simulation time. With the given angular velocity, $Energy\;input=2.045\times 10^{-18}$ J. The FiltratedWater at a specific time $t_{0}$ can be obtained by integrating the water flux shown in Figure 9a from initial time to $t_{0}$. For example, FiltratedWater=296.13 nm${}^{3}$ at the end of 30 ns. With relation: 1 J = 2.78 $\times 10^{-7}$ kWh, the energy efficiency is: 1.92 kWh/m${}^{3}$, which falls below the lower limit of traditional reverse osmosis RO (2∼5 kWh/m${}^{3}$) [34].

Our results indicate that this new nanopore membrane structure can act as a high-permeability and low energy consumption membrane for water filtration. We also provided guidelines for the design of macroscale nanoporous membrane co-axial centrifuge. Due to computational resource limitations, we only present a MD simulation of a nanoscale membrane centrifuge of 8 nm in diameter. However, the radius of the centrifuge may not affect the simulation result qualitatively, as long as the nanopores are created on the co-axial centrifuge wall, because we have no obvious size effect for such nanofluidic devices. From the theoretical perspective, if the size of the nanoporous membrane centrifuge reaches to macroscale, the critical angular velocity needed will be much smaller, which indicates that it is possible to fabricate such desalination centrifuge at engineering scale. Moreover, we can increase the water permeability by increasing the porosity of the membrane centrifuge, i.e., increasing the pore density on the membrane surface. Therefore, we propose a macroscale centrifuge that is decorated with nanoporous membrane to solve the scaling up problem existed in present nanomaterial based desalination technology.

## 4. Methods and Derivations

### 4.1. MD Simulation Details

The open source molecular dynamics software package, Gromacs [35], is used for all the MD calculations. The OPLS-aa force field [36] is used to describe bonded and nonbonded interactions between atoms, ions and molecules. The particle mesh Ewald method [37] is used for calculating long range electrostatic interactions, with cut-off distance of 0.8 nm, as is used in other similar MD simulations [25,27,38]. The SPC/E water model was used due to its advantage of simplicity, computational efficiency, and speed [39]. This water model is rigid and non-polarizable consisting of three interaction sites, located on the oxygen and hydrogen atomic centers, which shows the best overall agreement with experiments [40]. Periodic boundary conditions in all directions are employed. A total simulation time of 6 ns is conducted with data in the last 2 ns collected for data analysis. The integration time step is set to be 1 fs with a leap-frog algorithm [41]. The neighbor list is updated every time step to avoid intrinsic errors [42]. The MD system is first relaxed to minimize its free energy in a duration of 50 ps, followed by an NVT calculation by using the Nose-Hoover thermostat [43] to maintain a temperature at 298 K with damping coefficient of 0.1 ps. Then the non-equilibrium molecular dynamics simulation (NEMD) [29,44] featured with the rotating blade is then performed with the same Nose-Hoover thermostat still applied to the system. The rotating blade is prescribed to rotate around the central axis of the centrifuge rotator with a given angular velocity, and a tangential force perpendicular to the radial direction of the rotating blade may be exerted to each atom inside the centrifuge. Ten different values of the angular velocities ranging from 0.1 to 100 rad/ns are selected in the simulations. Even though the high angular velocities used in the simulations are almost practically impossible, however, since MD calculations are limited by their time scale, large angular velocities were required in order to satisfy the critical angular velocity condition to generate a sufficient pressure to balance the osmosis pressure. For practical desalination applications of the centrifuge, the readers are referred to the discussions on how to scale-up such desalination centrifuge.

#### 4.1.1. Feed Flow Motion Solution Derived from Fluid Dynamics Theory

From a continuum point of view, rotational fluid motion inside the model in Figure 2 can be considered as slipped Taylor-Couette flow in a confined nano-size space. The complex interactions of water molecules and hydrated ions near pores of the nanoporous membrane can be viewed as competitions of different forces: the centrifugal force due to internal rotation of the rotator, the osmosis force due to the solute concentration difference, and the Coriolis force due to the inertia effect. In addition to MD calculations, the critical centrifugal pressure, $P_{c}$, that balances the osmotic pressure can be derived from classical fluid dynamics.

According to water transport theory in membranes, the net water flux J (in unit cm/s) of a porous membrane may be expressed as: $J=N_{p}\left(L_{p}P_{\omega }-A_{c}V_{H_{2}O}\Delta C\right)$. Here, $N_{p}$ is the number of nano-sized pores at the wall of the nanoporous membrane. The first term on the right side is the water flux generated by centrifugal pressure (notated as the centrifugal pressure $P_{\omega }$ since it depends on the angular velocity ω in our model), with $L_{p}$ (in unit cm${}^{3}$/N·s) defined as hydraulic permeability. The second term is the water flux generated by osmotic pressure (notated as Π), with ΔC defined as the solute concentration gradient and $A_{c}$ the osmotic permeability (in unit cm/s). $V_{H_{2}O}$ is the molar volume of pure water. For seawater with salt concentration around 35 g/L at room temperature, ΔC=0.6 M and $V_{H_{2}O}=18.91$ cm${}^{3}$/mol.

Since the salt solution in the present study is relatively dilute, the osmotic pressure is related to ΔC through the linear equation: $\Pi =2R_{v}T\Delta C=3$ MPa ($R_{v}$ is the gas constant and T=300 K is the environmental temperature. The double value comes from pairs of Na${}^{+}$ and Cl${}^{-}$ ions in saltwater). The relation between osmotic permeability $A_{c}$ and hydraulic permeability $L_{p}$ can be derived according to the equilibrium condition (zero net water flux without centrifugal pressure: $J=0,P_{\omega }=0$): $A_{c}=\frac{R_{v}T}{V_{H_{2}O}}L_{p}$. Substituting this relation into the previous water flux equation, we can obtain the relation of hydraulic permeability as a function of net freshwater flux:

(1) $$J=L_{p}N_{p}\left(P_{\omega }-R_{v}T\Delta C\right).$$

In this work, the centrifugal pressure $P_{\omega }$ near pores of the membrane is generated by a rotational field, which can be derived by incorporating a classic Newtonian Couette flow under an angular velocity ω from the rotator. Consider the Navier-Stokes equation in fluid dynamics:

(2) $$\rho \left(\frac{\partial v}{\partial t}+v\cdot \left(\nabla \times v\right)\right)=-\nabla P+\mu \nabla^{2}v,$$

where ρ is the density and μ is dynamic viscosity. v and *P* are the velocity vector field and pressure field of the fluid respectively. Since our main focus in the current study is a first order approximation of pressure near the membrane wall, it is reasonable to assume that fluid motions in longitudinal and azimuthal directions are homogeneous without gradients of both velocities and pressure, which means

$$\frac{\partial v}{\partial z}=\frac{\partial v}{\partial \theta }=0,\;\;\mathrm{and}\;\;\frac{\partial P}{\partial z}=\frac{\partial P}{\partial \theta }=0.$$

In addition, since the angular velocity ω is large at the nanoscale, velocities in the longitudinal ($v_{z}$) and radial ($v_{r}$) directions are negligible in comparison with the velocity in azimuthal direction ($v_{\theta }$), which is usually two to three orders of magnitude larger. Therefore, the velocity and pressure can be approximated as: $v=[v_{z}=v_{r}=0,v_{\theta }\left(r\right)],P=P\left(r\right)$. With these assumptions and considering only the steady state solution, Equation (2) can be reduced to two ODEs:

(3) $$r^{2}\frac{d^{2}v_{\theta }}{dr^{2}}+r\frac{dv_{\theta }}{dr}-v_{\theta }=0,\;\;\;\;\;\;\;\;\;\frac{dP}{dr}=\rho \frac{v_{\theta }^{2}}{r}$$

The general solutions for the azimuthal velocity $v_{\theta }$ and pressure *P* of the fluid are:

(4) $$v_{\theta }=\frac{c_{1}}{r}+c_{2}r,\;\;P\left(r\right)=P\left(R_{i}\right)+\rho \left[\left(r^{2}-R_{i}^{2}\right)\left(\frac{c_{1}^{2}}{2R_{i}^{2}r^{2}}+\frac{c_{2}^{2}}{2}\right)+2c_{1}c_{2}\ln\frac{r}{R_{i}}\right]$$

where $c_{1}$ and $c_{2}$ are constants depending on boundary conditions (BCs). $R_{i}$ is the radius of inner-layer rotator where angular velocity ω is applied. $P(R_{i})$ is set to be the atmospheric pressure ($P\left(R_{i}\right)=P_{0}\approx 1$ Bar because we are assuming the centrifuge is in equilibrium with the external environment). Although the azimuthal velocities of the rotator at $r=R_{i}$ and the nanoporous membrane at $r=R_{o}$ are $\omega R_{i}$ and 0 respectively, these values are not necessarily equal to velocities of fluid near to the two boundaries. The slipping phenomenon for materials at nanoscale has been studied extensively [45], and different materials show different viscous and slipping properties [32,46]. Therefore, a detailed investigation is needed in MD calculation in order to quantify the slipping effect. Here a generalized situation is discussed:

Assuming the azimuthal velocities of fluid near the inner radius $r=R_{i}$ and the outer radius $r=R_{o}$ are $v_{\theta }\left(R_{i}\right)=\alpha_{i}R_{i}\omega$ and $v_{\theta }\left(R_{o}\right)=\alpha_{o}R_{i}\omega$ respectively. Where $\alpha_{i}$ and $\alpha_{0}$ are the slipping parameters of the inner and outer radius of the device, respectively. Note $\alpha_{o}\leq \alpha_{i}$ since no external force is applied on fluid between $R_{i}$ and $R_{o}$ and $v_{\theta }\left(r\right)$ will decay from $R_{i}$ to $R_{o}$ because of viscosity. Obviously, $\alpha_{i}=1,\alpha_{o}=0$ correspond to the classic no-slip BCs and $\alpha_{i}=\alpha_{o}=0$ correspond to the ideal-slip BCs. Therefore, the range of the two slipping parameters are: $0\leq \alpha_{i}\leq 1,\;0\leq \alpha_{o}\leq \alpha_{i}$. For energy efficiency, a non-slip BC for fluids near $r=R_{i}$ ($\alpha_{i}=1$) and an ideal-slip BC for fluids near $r=R_{o}$ ($\alpha_{o}=\alpha_{i}$) are preferred for best performance of the system, since $v_{\theta }\left(R_{i}\right)$ reaches maximal by the friction of the rotator and $v_{\theta }\left(R_{o}\right)$ is not affected by the friction of the static nanoporous membrane. Although neither the non-slip nor the ideal-slip conditions can be achieved for real materials, different materials can be selected to approach a limiting value.

The solutions of $v_{\theta }$ and *P* can be obtained as functions of $\alpha_{i}$ and $\alpha_{o}$ by substituting $v_{\theta }\left(R_{i}\right)$ and $v_{\theta }\left(R_{o}\right)$ into Equation (4). Here two types of BCs are listed explicitly for further discussion:

Boundary conditions case 1: $v_{\theta }\left(R_{i}\right)=\alpha_{i}R_{i}\omega ,v_{\theta }\left(R_{o}\right)=0$

(5) $$v_{\theta }\left(r\right)=\alpha_{i}\beta_{1}\left(\frac{R_{o}^{2}}{r}-r\right)\omega ,\;\;P\left(R_{o}\right)=P_{0}+\rho \alpha_{i}^{2}\beta_{1}^{2}\left(\frac{R_{o}^{2}+R_{i}^{2}}{2\beta_{1}}-2R_{o}^{2}\ln\frac{R_{o}}{R_{i}}\right)\omega^{2}$$

Boundary conditions case 2: $v_{\theta }\left(R_{i}\right)=v_{\theta }\left(R_{o}\right)=\alpha_{i}R_{i}\omega$

(6) $$v_{\theta }\left(r\right)=\alpha_{i}\beta_{2}\left(\frac{R_{i}R_{o}}{r}+r\right)\omega ,\;\;P\left(R_{o}\right)=P_{0}+\rho \alpha_{i}^{2}\beta_{2}^{2}\left(R_{o}^{2}-R_{i}^{2}+2R_{i}R_{o}\ln\frac{R_{o}}{R_{i}}\right)\omega^{2},$$

where

$$\beta_{1}=\frac{R_{i}^{2}}{R_{o}^{2}-R_{i}^{2}},\;\;\mathrm{and}\;\;\beta_{2}=\frac{R_{i}}{R_{o}+R_{i}}\;.$$

The pressure at the boundary of the cylinder $P(r=R_{o})$ can be solved because $P_{\omega }=P\left(R_{o}\right)$, after obtaining $\alpha_{i}$ from MD calculation. Here boundary conditions Case 1 and Case 2 correspond to the lower and upper bounds of $v_{\theta }$ and *P*. Therefore, these two types of BCs define the limiting performance that a given system can achieve. Moreover, a comparison between solutions from Equation (4) and MD results can quantify the derived analytical results from fluid dynamics for the nanofluidics systems.

#### 4.1.2. Comparisons between MD and Fluid Dynamics Theory

Within fluid dynamics theory, the expression of pressure in Equations (5) and (6) can be further simplified as

$$P_{\omega }=P\left(R_{o}\right)=P_{0}+\rho \alpha_{i}^{2}B\omega^{2}$$

where $B(R_{i},R_{o})$ is a function of geometry or the dimension (size) of the centrifuge that varies depending on different boundary condition, and it has the unit of $L^{2}$. In the case of the first boundary condition, we have

(7) $$B=\alpha_{i}^{2}\beta_{1}^{2}\left(\frac{R_{o}^{2}+R_{i}^{2}}{2\beta_{1}}-2R_{o}^{2}\ln\frac{R_{o}}{R_{i}}\right)$$

and in the case of the second boundary condition, we have

(8) $$B=\alpha_{i}^{2}\beta_{2}^{2}\left(R_{o}^{2}-R_{i}^{2}+2R_{i}R_{o}\ln\frac{R_{o}}{R_{i}}\right)\;.$$

Thus, the parameter *B* is proportional to the radius of the centrifuge, i.e., $B∼R_{o}^{2}$.

The centrifugal pressure $P_{\omega }$ can be substituted into Equation (1) to obtain the expression of net water flux *J* as a function of angular velocity ω shown below. Meanwhile, a minimum or critical angular velocity $\omega_{cr}$ can be derived by setting J=0, i.e.,

(9) $$J=L_{p}N_{p}\left[\left(P_{0}-R_{v}T\Delta C\right)+\rho \alpha_{i}^{2}B\omega^{2}\right]\;.$$

Within MD calculations, slipping parameter $\alpha_{i}$ can be obtained by investigating the velocity profiles of water molecules. The net water flux $J^{{}^{\prime }}$ can be obtained by simply counting the number of water molecules ($N_{H_{2}O}$) passing through the nanoporous membrane within the simulation time,

$$J^{{}^{\prime }}=\frac{N_{H_{2}O}}{N_{A}V_{H_{2}O}A_{m}\Delta t},$$

where $N_{A}$ is Avogadro’s number; $A_{m}$ is the area of the membrane, and Δt is the simulation time). Stable $J^{{}^{\prime }}$ can be obtained for each ω when Δt is big enough. The relationship between $J^{{}^{\prime }}$ and angular velocity ω can be plotted and directly compared with Equation (9). The critical angular velocity, $\omega_{cr}$, derived from Equation (9),

$$\omega_{cr}=\sqrt{\frac{R_{v}T\Delta C-P_{0}}{\rho \alpha_{i}^{2}B}},\;\;\mathrm{where}\;B∼R_{o}^{2}$$

can also be compared with the applied ω in the MD calculation when $J^{{}^{\prime }}=0$. Velocities obtained from MD calculations can also be compared to the analytical velocity result, $v_{\theta }\left(r\right)$, derived from Equation (4). However, the comparison of the centrifugal pressure between MD calculations and Equations (5) and (6) needs further analysis, because the pressure defined in the MD simulation is for the whole system, instead of the value at $r=R_{o}$. Nonetheless, the original definition of pressure (force per area) can be used to derive the centrifugal pressure $P_{\omega }$ based on MD theory, with the expression

$$P_{\omega }=\frac{\sum_{i=1}^{N_{H_{2}O}}F_{ir}}{A_{m}}$$

where $F_{ir}$ is the radial component of the force vector exerted on water molecule *i*. Here, the pressure calculated from the sum of the total force experienced on each water molecule in the direction of the membrane divided by the area of the membrane is purely based on Newton’s laws of motion. By comparing the centrifugal pressure $P_{\omega }$ from MD calculations with values obtained from Equations (5) and (6), it is possible to investigate whether or not $P_{\omega }$ at micro-scale is still within the bounds defined at macro-scale.

In general, by comparing results from two independent theories, it would be possible to analyze the size effect and discuss how the performance of the system varies if the size of the system is scaled up from micro-scale to macro-scale.

## Figures and Tables

**Figure A1:**
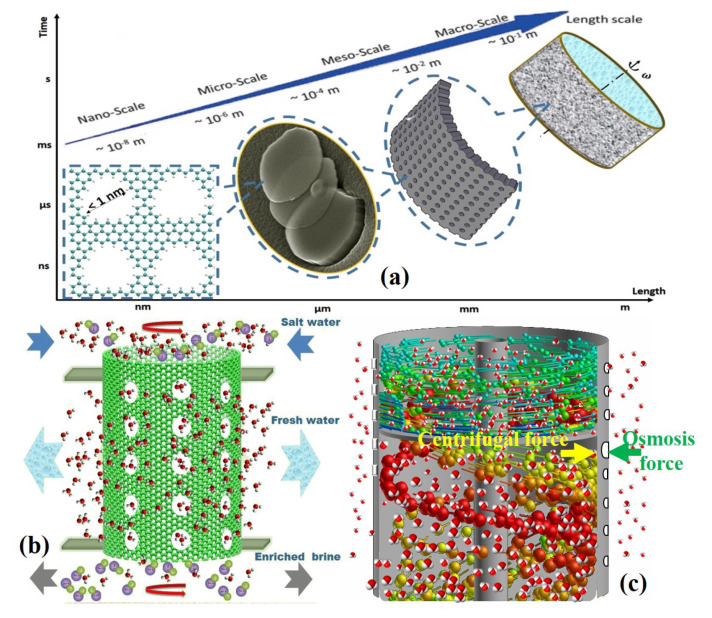
Schematic illustration on how to scale-up a nano-porous rotating membrane to macroscale and its macroscale fabrication concept. (**a**) Multiscale porous membrane structure, (**b**) Rotating porous membrane, and (**c**) A rotator-generated centrifugal fluid motion of the feed solution (seawater in RO).

## Appendix A. Fluid Dynamics Solutions

### Appendix A.1. Derivation of the Simplified Governing Equation (3) from the Navier-Stokes Equation

The θ-component of the N-S equation in Cylindrical Coordinates (r,θ,z) reads as follows,

(A1) $$\begin{array}{c} \rho \left(\frac{\partial \nu_{\theta }}{\partial t}+\nu_{r}\frac{\partial \nu_{\theta }}{\partial r}+\frac{\nu_{\theta }}{r}\frac{\partial \nu_{\theta }}{\partial \theta }+\frac{\nu_{r}\nu_{\theta }}{r}+\nu_{z}\frac{\partial \nu_{\theta }}{\partial z}\right)\\ =-\frac{1}{r}\frac{\partial P}{\partial \theta }+\mu \left(\frac{1}{r}\frac{\partial }{\partial r}\left(r\frac{\partial \nu_{\theta }}{\partial r}\right)-\frac{\nu_{\theta }}{r^{2}}+\frac{1}{r^{2}}\frac{\partial^{2}\nu_{\theta }}{\partial \theta^{2}}+\frac{2}{r^{2}}\frac{\partial \nu_{r}}{\partial \theta }+\frac{\partial^{2}\nu_{\theta }}{\partial z^{2}}\right)\;. \end{array}$$

The r-component of N-S equation in Cylindrical Coordinates (r,θ,z):

(A2) $$\begin{array}{c} \rho \left(\frac{\partial \nu_{r}}{\partial t}+\nu_{r}\frac{\partial \nu_{r}}{\partial r}+\frac{\nu_{\theta }}{r}\frac{\partial \nu_{r}}{\partial \theta }-\frac{\nu_{\theta }^{2}}{r}+\nu_{z}\frac{\partial \nu_{r}}{\partial z}\right)\\ =-\frac{\partial P}{\partial r}+\mu \left(\frac{1}{r}\frac{\partial }{\partial r}\left(r\frac{\partial \nu_{r}}{\partial r}\right)-\frac{\nu_{r}}{r^{2}}+\frac{1}{r^{2}}\frac{\partial^{2}\nu_{\theta }}{\partial \theta^{2}}-\frac{2}{r^{2}}\frac{\partial v_{\theta }}{\partial \theta }+\frac{\partial^{2}\nu_{r}}{\partial z^{2}}\right) \end{array}$$

In the ensuing derivation, the following assumptions are made, as discussed in the main text of the paper,

1. Steady state:
  $$\frac{\partial }{\partial t}=0;$$
2. Homogeneous in θ and *z* direction (No gradient):
  $$\frac{\partial }{\partial z}=\frac{\partial }{\partial \theta }=0;$$
3. Velocity in θ direction is much higher than *z* and *r* directions, i.e., $\nu_{\theta }>>\nu_{z},\nu_{r}$, therefore it is assumed that $\nu_{z}=\nu_{r}\approx 0$.

Considering these assumptions, from Equation (A1), we obtain,

(A3) $$0=\mu \left(\frac{1}{r}\frac{\partial }{\partial r}\left(r\frac{\partial \nu_{\theta }}{\partial r}\right)-\frac{\nu_{\theta }}{r^{2}}\right)\;.$$

Considering these assumptions in Equation (A2), we then obtain,

(A4) $$-\rho \frac{\nu_{\theta }^{2}}{r}=-\frac{\partial P}{\partial r}\;.$$

Equations (A3) and (A4) can be cast as Equation (A5), which is the same as Equation (3) in the main text,

(A5) $$r^{2}\frac{d^{2}\nu_{\theta }}{dr^{2}}+r\frac{d\nu_{\theta }}{dr}-\nu_{\theta }=0,\;\;\;\;\;\;\frac{dP}{dr}=\rho \frac{\nu_{\theta }^{2}}{r}\;.$$

### Appendix A.2. Derivation of General Solutions (4) from N-S Equation

The general solution of $\nu_{\theta }$ in Equation (A5) is,

(A6) $$\nu_{\theta }=\frac{c_{1}}{r}+c_{2}r$$

where the constants $c_{1}$ and $c_{2}$ are defined from the boundary conditions.

Substituting $\nu_{\theta }$ into equation,

$$\frac{dP}{dr}=P\frac{\nu_{\theta }^{2}}{r},$$

and integrating over the whole domain leads to,

(A7) $$\begin{array}{cc} P(r^{\prime }) & =P\left(R_{i}\right)+\int_{R_{i}}^{r^{\prime }}\rho \frac{\nu_{\theta }^{2}}{r}dr\\ & =P\left(R_{i}\right)+\rho \int_{R_{i}}^{r^{\prime }}\frac{1}{r}{\left(\frac{c_{1}}{r}+c_{2}r\right)}^{2}dr\\ & =P\left(R_{i}\right)+\rho \int_{R_{i}}^{r^{\prime }}\left(\frac{c_{1}^{2}}{r^{3}}+\frac{c_{1}c_{2}}{r}+c_{2}^{2}r\right)dr\\ & =P\left(R_{i}\right)+\rho \int_{R_{i}}^{r^{\prime }}d\left(-\frac{c_{1}^{2}}{2r^{2}}+2c_{1}c_{2}ln\left(r\right)+\frac{1}{2}c_{2}^{2}r^{2}\right)\\ & =P\left(R_{i}\right)+\rho \left(-\frac{c_{1}^{2}}{2r^{\prime 2}}+\frac{c_{1}^{2}}{2R_{i}^{2}}+2c_{1}c_{2}ln\left(\frac{r^{\prime }}{R_{i}}\right)+\frac{1}{2}c_{2}^{2}\left(r^{\prime }{}^{2}-r_{i}^{2}\right)\right)\\ & =P\left(R_{i}\right)+\rho \left(\left(r^{\prime }{}^{2}-R_{i}^{2}\right)\left(\frac{c_{1}^{2}}{2r^{\prime }{}^{2}R_{i}^{2}}+\frac{c_{2}^{2}}{2}\right)+2c_{1}c_{2}ln\left(\frac{r^{\prime }}{R_{i}}\right)\right). \end{array}$$

### Appendix A.3. Solution of Equation (4) under Two Special Boundary Conditions Equations (5) and (6)

**I.** From the boundary conditions: $\nu_{\theta }\left(R_{i}\right)=\alpha_{i}R_{i}\omega_{i}$ and $\nu_{\theta }\left(R_{0}\right)=0$, we have

(A8) $$\left\{\begin{array}{cc} \nu_{\theta }\left(R_{i}\right)=\frac{c_{1}}{R_{i}}+c_{2}R_{i}=\alpha_{i}R_{i}\omega , & \\ \;\nu_{\theta }\left(R_{0}\right)=\frac{c_{1}}{R_{0}}+c_{2}R_{0}=0, & \end{array}\right.$$

Then $c_{1}$ and $c_{2}$ can be solved as,

(A9) $$\left\{\begin{array}{cc} c_{1}=\frac{\alpha_{i}R_{i}^{2}R_{0}^{2}}{R_{0}^{2}-R_{i}^{2}}\omega =\alpha_{i}\beta_{1}R_{0}^{2}\omega , & \\ \;c_{2}=\frac{\alpha_{i}R_{i}^{2}}{R_{0}^{2}-R_{i}^{2}}\omega =-\alpha_{i}\beta_{1}\omega , & \;. \end{array}\right.$$

where

$$\beta_{1}=\frac{R_{i}^{2}}{R_{0}^{2}-R_{i}^{2}}\;.$$

Then $\nu_{\theta }\left(r\right)$ can be rewritten as,

(A10) $$\nu_{\theta }\left(r\right)=\frac{\alpha_{i}R_{i}^{2}R_{0}^{2}}{R_{0}^{2}-R_{i}^{2}}\frac{\omega }{r}-\frac{\alpha_{i}R_{i}^{2}}{R_{0}^{2}-R_{i}^{2}}\omega r=\alpha_{i}\beta_{1}\left(\frac{R_{0}^{2}}{r}-r\right)\omega$$

When *r* = $R_{0}$, the pressure $P_{\omega }$ is given as,

(A11) $$\begin{array}{cc} P_{w} & =P\left(R_{i}\right)+\rho \left(\left(r_{0}^{2}-R_{i}^{2}\right)\left(\frac{c_{1}^{2}}{2r_{0}^{2}R_{i}^{2}}+\frac{c_{2}^{2}}{2}\right)+2c_{1}c_{2}ln\left(\frac{R_{0}}{R_{i}}\right)\right)\\ & =P\left(R_{i}\right)+\rho \alpha_{i}^{2}\beta_{1}^{2}\left(\frac{R_{0}^{2}+R_{i}^{2}}{2\beta_{1}}-2R_{0}ln\left(\frac{R_{0}}{R_{i}}\right)\right)\omega^{2}\;. \end{array}$$

**II.** For the boundary conditions: $\nu_{\theta }\left(R_{i}\right)$ = $\nu_{\theta }\left(R_{0}\right)$ = $\alpha_{i}R_{i}\omega$, we have,

(A12) $$\left\{\begin{array}{cc} \nu_{\theta }\left(R_{i}\right)=\frac{c_{1}}{R_{i}}+c_{2}R_{i}=\alpha_{i}R_{i}\omega , & \\ \;\nu_{\theta }\left(R_{0}\right)=\frac{c_{1}}{R_{0}}+c_{2}R_{0}=\alpha_{i}R_{i}\omega , & \end{array}\right.$$

Then $c_{1}$ and $c_{2}$ can be solved as,

(A13) $$\left\{\begin{array}{cc} c_{1}=\frac{\alpha_{i}R_{i}^{2}R_{0}^{2}}{R_{0}+R_{i}}\omega =\alpha_{i}\beta_{2}R_{0}R_{i}\omega , & \\ \;c_{2}=\frac{\alpha_{i}R_{i}}{R_{0}+R_{i}}\omega =\alpha_{i}\beta_{2}\omega , & \end{array}\right.$$

where

$$\beta_{2}=\frac{R_{i}}{R_{i}+R_{o}}\;.$$

Then $\nu_{\theta }\left(r\right)$ can be rewritten as,

(A14) $$\nu_{\theta }\left(r\right)=\alpha_{i}\beta_{2}\left(\frac{R_{0}R_{i}}{r}+r\right)\omega$$

When *r* = $R_{0}$, the pressure $P_{\omega }$ is given as,

(A15) $$\begin{array}{cc} P_{w} & =P\left(R_{i}\right)+\rho \left(\left(R_{0}^{2}-R_{i}^{2}\right)\left(\frac{\alpha_{i}^{2}\beta_{2}^{2}R_{0}^{2}R_{i}^{2}\omega^{2}}{2R_{0}^{2}R_{i}^{2}}+\frac{\alpha_{i}^{2}\beta_{2}^{2}\omega^{2}}{2}\right)+2\alpha_{i}^{2}\beta_{2}^{2}R_{0}R_{i}\omega^{2}ln\left(\frac{R_{0}}{R_{i}}\right)\right)\\ & =P\left(R_{i}\right)+\rho \alpha_{i}^{2}\beta_{2}^{2}\omega^{2}\left(\left(R_{0}^{2}-R_{i}^{2}\right)+2R_{0}R_{i}ln\left(\frac{R_{0}}{R_{i}}\right)\right) \end{array}$$

### Appendix A.4. Derivation of the Critical Angular Velocity, ω_cr_:

Consider the fact that

(A16) $$\begin{array}{cc} J & =L_{p}N_{p}\left(P_{w}-R_{0}T\Delta C\right)\\ & =L_{p}N_{p}\left(P_{0}+\rho \alpha_{i}^{2}Bw^{2}-R_{\nu }T\Delta C\right)\;. \end{array}$$

When $J(\omega_{cr}$) = 0, it leads to,

(A17) $$\rho \alpha_{i}^{2}B\omega_{cr}^{2}=R_{\nu }T\Delta C-P_{0}.$$

Finally, $\omega_{cr}$ can be solved as follows,

(A18) $$\omega_{cr}=\sqrt{\frac{R_{\nu }T\Delta C-P_{0}}{\rho \alpha_{i}^{2}B}}\;.$$

where $B=B(R_{i},R_{o})$ is a function of dimension of the centrifuge that varies under different boundary conditions, and it has units of $L^{2}$. For the first type of boundary conditions, we can derive that

(A19) $$B=\alpha_{i}^{2}\beta_{1}^{2}\left(\frac{R_{o}^{2}+R_{i}^{2}}{2\beta_{1}}-2R_{o}^{2}\ln\frac{R_{o}}{R_{i}}\right)=\alpha_{i}^{2}\beta_{1}^{2}\left(\frac{1+r^{2}}{2\beta_{1}}+2\ln r\right)R_{o}^{2}$$

and the second set of boundary conditions, we have,

(A20) $$B=\alpha_{i}^{2}\beta_{2}^{2}\left(R_{o}^{2}-R_{i}^{2}+2R_{i}R_{o}\ln\frac{R_{o}}{R_{i}}\right)=\alpha_{i}^{2}\beta_{1}^{2}\left(\frac{1-r^{2}}{2\beta_{1}}-2\ln r\right)R_{o}^{2}$$

where $0<r=R_{i}/R_{o}\leq 1$. Therefore, in general, $B∼C\left(\alpha_{i},\beta_{i},r\right)R_{o}^{2}$, and *C* is a function of the inner radius of the centrifuge and the boundary condition of the centrifuge.

## Appendix B. Scale-Up Idea to Fabricate a Macroscale Nanoporous Rotating Centrifuge

In Appendix B, we display a graphic illustration on how to scale up the proposed rotational nanofluidic device with nanoporous membrane wall. The illustration demonstrate both the scale-up concept as well as the detailed fabrication steps in Figure A1. The key to fabricate such scale up rotating nano-porous graphene membrane centrifuge is to create a multiscale porous structure. The first step is to drill micron scale tine holes on the metal wall or other structural material wall that can provide structure support for a macroscale system with its dimension $R_{o}>1$ m. The second step is to paste small nano-porous graphene patches to cover those micron scale holes as illustrated in Figure A1. By doing so, we can achieve:

1. having a nano-porous membrane on the wall of the centrifuge;
2. having a strong wall structure to support macroscale loads such as overall pressure under working conditions;
3. having a realistic critical angular velocity of a few hundreds of radian per second to generate a centrifugal pressure $P_{\omega }$ that is greater than the osmosis pressure.
